# A Novel Diagnostic Method for Invasive Fungal Disease Using the Factor G Alpha Subunit From *Limulus polyphemus*

**DOI:** 10.3389/fmicb.2021.658144

**Published:** 2021-06-28

**Authors:** Fang Cui, Peng Luo, Yao Bai, Jiangping Meng

**Affiliations:** ^1^Department of Laboratory Medicine, The First Affiliated Hospital of Chongqing Medical University, Chongqing, China; ^2^Assisted Reproductive Center, Department of Obstetrics and Gynecology, The First Affiliated Hospital of Chongqing Medical University, Chongqing, China

**Keywords:** invasive fungal disease, G Factor α subunit, sandwich method, diagnosis, methodology

## Abstract

Deaths due to invasive fungal disease (IFD) have been increasing every year. Early and rapid detection is important to reduce the mortality rate associated with IFD. In this study, we explored a novel diagnostic method for detecting IFD, which involves the G Factor α subunit (GFαSub) from *Limulus polyphemus*. The GFαSub double-sandwich method was developed to detect (1,3)-β-D-glucans in human serum using purified GFαSub and horseradish peroxidase-labeled GFαSub. The GFαSub double-sandwich method and the G test were performed and compared. Using GFαSub sequence analysis, the expression plasmid pET30a-GFαSub252-668 was synthesized, and GFαSub252-668 was expressed and purified *via* isopropyl-β-d-thiogalactoside induction and nickel-nitrilotriacetic acid affinity. The optimization method was established *via* the orthogonal method. Using this method, the sera of 36 patients with IFD and 92 volunteers without IFD underwent detection, and the receiver operating characteristic curve of the GFαSub252-668 double-sandwich method was described. The sensitivity and specificity of the GFαSub252-668 double-sandwich method were 91.67 and 82.61%, respectively, and there was good correlation with the G test for the serum specimens of 36 patients with pulmonary IFD (*R*^2^ = 0.7592). In conclusion, our study suggests that the GFαSub252-668 double-sandwich method was satisfactory at detecting IFD cases. This method can be promoted and further developed as a novel method for diagnosing IFD.

## Introduction

The incidence rate of invasive fungal infections (IFD) in the general population has remained static year-on-year; however, with respect to at-risk individuals, the incidence rate has risen every year. Although most fungi do not cause infection in healthy individuals, IFD most commonly occurs in immunocompromised persons and those with autoimmune disorders (Enoch et al., 2017; Posch et al., 2017; Scriven et al., 2017). Lungs are the most common site of IFD, and it is difficult to differentiate pulmonary IFD from bacterial infections because of their similar clinical presentations (Schelenz et al., 2015; Acharige et al., 2018; Ajmal et al., 2018). The clinical consequences of IFD are severe; therefore, the differential diagnosis of IFD is critical for guiding clinical treatment. To identify IFD, imaging and laboratory examinations are employed (Calitri et al., 2017; Calley and Warris, 2017; Katragkou et al., 2017). Other examinations include fungal smears, cultures, serological tests, and the G test. Despite the ability of these tests to accurately detect IFD, they have certain limitations. Fungal cultures generally require 2 days, whereas fungal smears only take few minutes but have high false-negative rates. Moreover, serological tests cannot distinguish current infections from past infections, particularly if only one test is utilized. The antifungal antibody or antigen also depends on a person’s immunity level. Polymerase chain reaction (PCR) testing is sensitive but cannot identify the proliferation and survival of the fungus. In our clinic, the G test is used to detect and diagnose IFD. The principle behind this method is that (1,3)-β-D-glucan in the fungal cell wall can activate the G factor to catalyze the coagulation cascade in the *Limulus* plasma; the subsequent conversion of fibrinogen into fibrin is detected *via* a dynamic turbidimeter.

During IFD, phagocytes consume fungal spores and release (1,3)-β-D-glucan, a highly abundant fungal cell wall component, into the circulatory system. Measuring (1,3)-β-D-glucan levels, in combination with clinical signs and symptoms, might aid in diagnosing IFD (Shi et al., 2016; Verma et al., 2019). Hence, a method with high sensitivity that can accurately diagnose IFD should be developed. Because (1,3)-β-D-glucan is a sugar with varying degrees of polymerization, it may be difficult to obtain a specific antibody for this purpose. Glucan-binding proteins (GBPs) are natural proteins that are bound to glucan and are found in animals, plants, bacteria, and even in humans. In our previous study, we verified the binding forces of several GBPs for detecting (1,3)-β-D-glucan. The results showed that the clotting factor G alpha subunit (GFαSub) from *Limulus polyphemus* was better detected than other GBPs. In addition, based on the structural domains and functions of GfαSub, its truncated version can be an excellent ligand for research and clinical examination.

In the present study, we used the truncated GfαSub to quantify (1,3)-β-D-glucan in clinical samples (Ueda et al., 2009; Adachi et al., 2019). The study was designed to utilize the high specificity and sensitivity of the immunoassay to improve the clinical and laboratory diagnosis of IFD.

## Materials and Methods

### Subjects

We selected 36 patients with deep respiratory tract fungal infections from January 2017 to December 2019 and classified them as the positive group. In total, 92 volunteers were assigned to the negative group: 52 had bacterial lung infection, which was confirmed by the clinical laboratory, and 40 were healthy volunteers without any infection.

Invasive fungal disease was diagnosed as per the guidelines of the European Organization for Research and Treatment of Cancer/Mycoses Study Group (EORTC/MSG) (Donnelly et al., 2020). The inclusion criteria for patients with IFD were as follows: (1) a recent history of IFD diagnosis, treatment, radiotherapy, chemotherapy, or hormone use accompanied by fever, cough, shortness of breath, and other respiratory symptoms; (2) positive sputum fungal culture and/or sputum fungal smear; (3) positive G test; and (4) detection of elevated levels of interleukin-6, procalcitonin, or both (Patterson et al., 2016). Patients with pulmonary bacterial infection were those who showed pathogenic gram-negative bacilli or gram-positive cocci on culture and had been treated effectively as per the information on drug sensitivity. However, patients with concurrent pulmonary bacterial and fungal infections were excluded. The clinical information of the 36 patients with IFD is listed in Table 1. A total of 128 subjects were included in this study, and their physiological information is shown in Table 2. In a fasting state, 4 mL whole blood was obtained in a procoagulant tube from each participant. Serum was separated and stored at −80°C until use. (1,3)-β-D-glucan levels were measured using the G test with commercial kits (Zhanjiang A&C Biological Co., Ltd) and the in-house developed GFαSub double-sandwich method.

**TABLE 1:** The basic information of 36 IFD patients.

Items		Total	Molds	Yeasts
		36	(*n* = 6)	(*n* = 30)
Sputum smear		Positive results (*n*)	3	26
Sputum culture			6	30
Blood culture			1	2
G-test			5	30
Imaging			6	30
Co-infection	G^–^b		5	26
	G^+^c		–	3

**TABLE 2:** The comparison of the basic information of the 128 subjects.

	Pulmonary with IFD (*n* = 36)	Pulmonary with BI (*n* = 52)	Healthy donors (*n* = 40)	*F, t, χ*^2/^*p*
Gender (M/F)	7/2	9/4	3/1	0.3348, 0.8459
Age (year)	46.9 ± 15.2	49.1 ± 20.8	48.3 ± 17.3	0.1543, 0.8572
BMI	23.2 ± 9.1	22.6 ± 7.2	28.3 ± 8.6	2.554, 0.2789
Yeast/Mould	5/1	–	–	–
G^–^b/G^+^c	31/3	21/5	–	–

*M/F, male and female; BMI, Body Mass Index; G^–^b/G^+^c, gram-negative bacillus and gram-positive cocci; BI, bacterial infection.*

### Materials

The expression plasmid of pET30a-GFαSub252-668 was synthesized by Shanghai Biotechnology Company (China), and *Escherichia coli* BL21 (DE3) was maintained in our laboratory. (1,3)-β-D-glucan was purchased from Elicityl Co., Ltd., (France), and restriction endonucleases and digestion buffer were purchased from Fermentas Co., Ltd (Canada). The monoclonal antibody of anti-His tag was purchased from Beijing Zhongshan Jinqiao Biological Co., Ltd (China), whereas the horseradish peroxidase (HRP)-labeling kit, protein-free blocking solution, 3,3′,5,5′-tetramethylbenzidine chromogenic solution, and termination solution were purchased from Jinan Taitianhe Biological Co., Ltd (China). The nickel-nitrilotriacetic acid (Ni-NTA) purification column was purchased from Qiagen (Germany). The sodium dodecyl sulfate-polyacrylamide gel electrophoresis (SDS-PAGE) preparation kit and kanamycin were purchased from Solibao Biological Company (China). Protein and DNA ladder markers were purchased from ThermoFisher (United States). Phenylmethylsulfonyl fluoride (PMSF), protease inhibitor cocktail, and isopropyl-β-D-thiogalactoside (IPTG) were purchased from Aladdin (China). The remaining chemical reagents were purchased from Sinopharm Chemical Group Corporation (China).

### Characterization of the GFαSub252-668 Expression Plasmid

Based on the clone site information about the constructed plasmid pET30a-GFαSub252-668, a double restriction enzyme digestion using NedI and *Hin*dIII was performed for characterization. The reaction system was as follows: 10 mL of plasmid, 1 mL each of NedI and *Hin*dIII, 2 mL of 10 × buffer, and 6 mL of deionized water. The system was placed in a water bath at 37°C for 30 min, and then heated at 95°C for 5 min to terminate the reaction. Products were identified by electrophoresis on 1% agarose gels.

### Expression and Purification of GFαSub252-668

The plasmid pET30a-GFαSub252-668 was transfected into *E. coli* BL21 (DE3). Next, the strain underwent heat shock at 42°C for 90 s. Positive clones were screened on kanamycin-resistant Luria–Bertani (LB) solid medium and then inoculated into LB liquid medium. IPTG was added when the turbidity value of optical density (OD)_600 nm_ reached 0.8 with a final concentration of 0.8 mM. The strains were then induced overnight at room temperature (25°C). The strains were subsequently collected by centrifugation at 10,000 × *g* for 30 min and rinsed twice with sterile pre-cooled phosphate-buffered saline (PBS). Strains were then treated by repeated freezing (−80°C) and thawing followed by another ultrasonic fragmentation for 10 min. Finally, the supernatants and precipitates were collected separately by centrifugation at 10,000 × *g* for 30 min. SDS-PAGE was performed, and the target protein was identified using Coomassie brilliant blue staining. The lysed supernatants and Ni-NTA purification column were placed on a 4°C mixer and continuously mixed for 2 h at room temperature. PMSF and protease inhibitor cocktail were added during the mixing. Then, the column was subjected to washing and eluting with various concentrations of imidazole buffer. The eluent was identified again by SDS-PAGE and stained using Coomassie brilliant blue staining.

### Establishing the Double-Sandwich Testing System

The purified protein of GFαSub was quantified and then labeled using an HRP-labeling kit as per its molar concentration. Next, a double-sandwich testing method for GFαSub252-668 was established *via* the chessboard method: the measurement was performed by coating with various concentrations of GFαSub252-668, and GFαSub252-668 was then labeled with HRP. PBS containing 10% dimethyl sulfoxide (DMSO) served as the negative control, while (1,3)-β-D-glucan diluted in 200 pg/mL of PBS containing 10% DMSO served as the positive control. After determining the optimal coating and detection concentrations, the sera of healthy controls and the final concentration of 200 pg/mL (1,3)-β-D-glucan were used as negative and positive samples, respectively, and six multiple wells were set for each group to verify testing efficiency.

### Comparison of Testing Efficiency Between the Double-Sandwich and G Test Methods

We performed a double-sandwich assay of GFαSub252-668 in 128 human serum samples. Meanwhile, 0, 50, 100, 200, and 400 pg/mL of (1,3)-β-D-glucan were used for quantitative standard curves. We calculated correlations between the levels of measured (1,3)-β-D-glucan using the double-sandwich and G test methods.

### Statistical Analysis

Statistical analyses were performed using SPSS 15.0. The comparison of quantitative data between the two groups was determined using a *t*-test, while differences in the constituent ratio among various groups were compared *via* the chi-square test. Correlation analysis between the two groups of quantitative data was performed using Spearman’s method. All *p*-values were considered statistically significant if *p* < 0.05.

## Results

### Establishing the GFαSub252-668 Double-Sandwich Method

GFαSub252-668 was inserted into pET30a, and the presence of the inserted gene was confirmed by restriction digestion (Supplementary Figure 1). The identity of the inserted gene was verified by sequencing. The expression of recombinant GFαSub252-668 was induced by IPTG (Supplementary Figure 2), purified using the Ni-NTA column (Supplementary Figure 3), and detected with the anti-His tag monoclonal antibody (Supplementary Figure 4).

**FIGURE 1:**
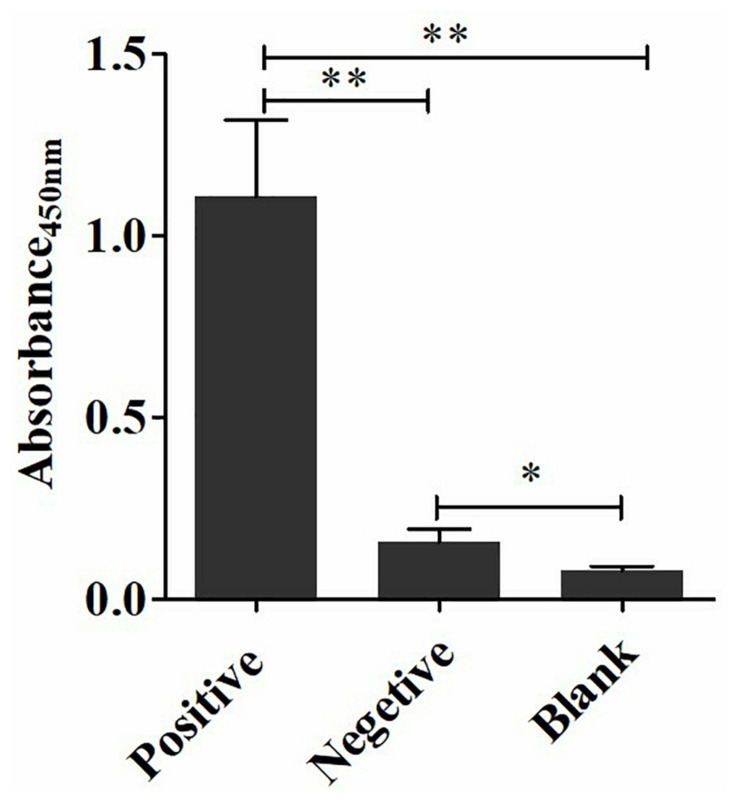
Determination of (1,3)-β-D-glucan serum levels using the GFαSub252-668 double-sandwich method. * *P* < 0.05, ** *P* < 0.01.

The coating concentration of GFαSub252-668 and the testing concentration of GFαSub252-668-HRP were optimized using the orthogonal method with the uniform conditions of incubation, blocking, rinsing, and coloration. Based on the value of absorbance in the 450 nm wavelength and the ratio of absorbance positive/absorbance negative, the maximum value of the ratio of absorbance positive/absorbance negative was 5.064 when the coating concentration of GFαSub252-668 and working concentration of GFαSub252-668-HRP were 0.8 and 1.6 ng/mL, respectively (Table 3). To verify the influence of the matrix effect on the method, healthy human serum containing 200 pg/mL of (1,3)-β-D-glucan was considered as a positive sample and healthy human serum was taken as a negative sample. The GFαSub252-668 double-sandwich method was used for testing, and the OD ratio for positive to negative samples was approximately 6, which clearly distinguished the negative and positive samples (Figure 1).

**TABLE 3:** Determination of the working concentrations of the reagent for coating and testing in the GFαSub252-668 double sandwich method using the orthogonal method.

Coating (ng/mL)	Testing (ng/mL) (Absorbance of Positive team)					Testing (ng/mL) (Absorbance of Negative team)				
	0.1	0.2	0.4	0.8	1.6	0.1	0.2	0.4	0.8	1.6
0.1	0.289	0.391	0.466	0.583	0.636	0.087	0.102	0.116	0.122	0.146
0.2	0.339	0.447	0.529	0.613	0.738	0.107	0.125	0.131	0.148	0.182
0.4	0.395	0.501	0.627	0.706	0.892	0.133	0.154	0.161	0.189	0.205
0.8	0.413	0.613	0.736	0.866	**1.094**	0.169	0.181	0.196	0.209	**0.216**
1.6	0.484	0.705	0.843	1.003	1.246	0.205	0.213	0.227	0.235	0.285

*Underlined number stands for optimum concentration of coating and testing, MAX = Abs_P_/Abs_N_.*

### Comparison of the Clinical Testing Efficiency Between the GFαSub252-668 Double-Sandwich and G Test Methods

**FIGURE 2:**
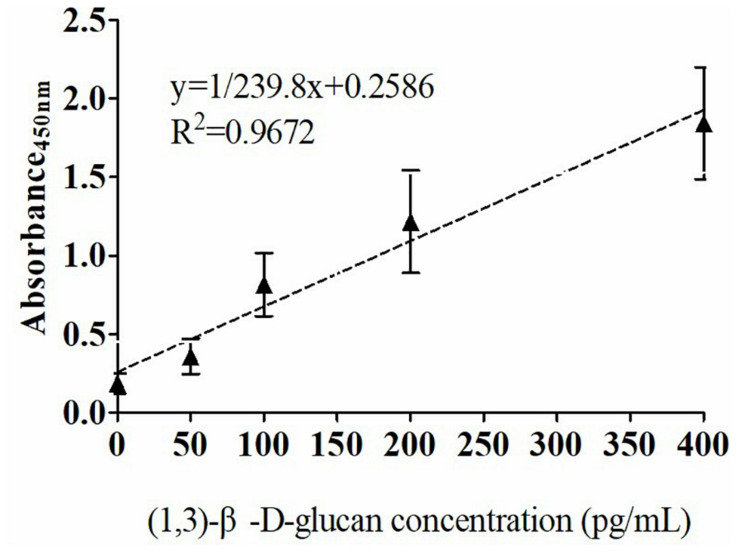
Standard curve of the GFαSub252-668 double-sandwich method for determining the level of (1,3)-β-D-glucan in serum.

**FIGURE 3:**
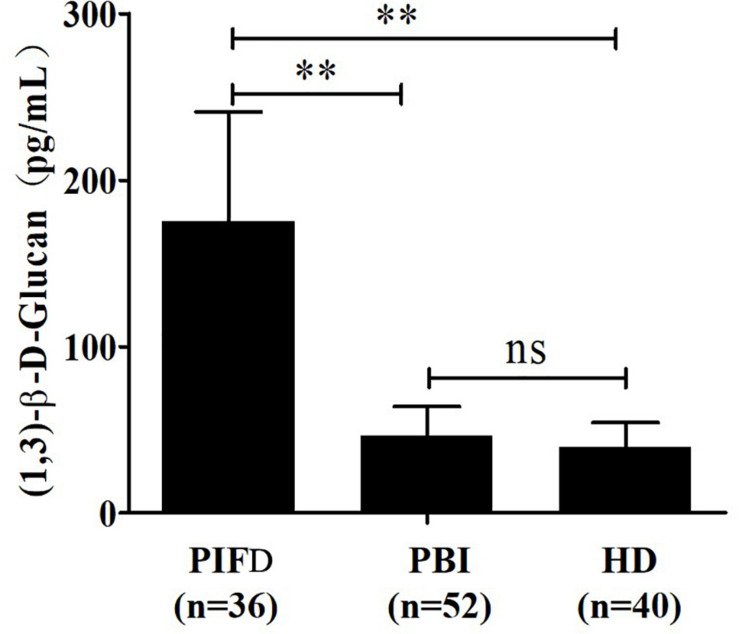
Determination of serum (1,3)-β-D-glucan levels in 128 subjects using the GFαSub252-668 double-sandwich method. PIFD, pulmonary invasive fungal disease; PBI, pulmonary bacterial infection; HD, healthy donors; ns, not significant; **p < 0.001. IFD group vs. bacterial infection group: *t* = 13.47, *p* < 0.001; IFD group vs. healthy group: *t* = 12.7, *p* < 0.001; bacterial infection group vs. healthy group: *t* = 1.983, *p* = 0.0504.

**FIGURE 4:**
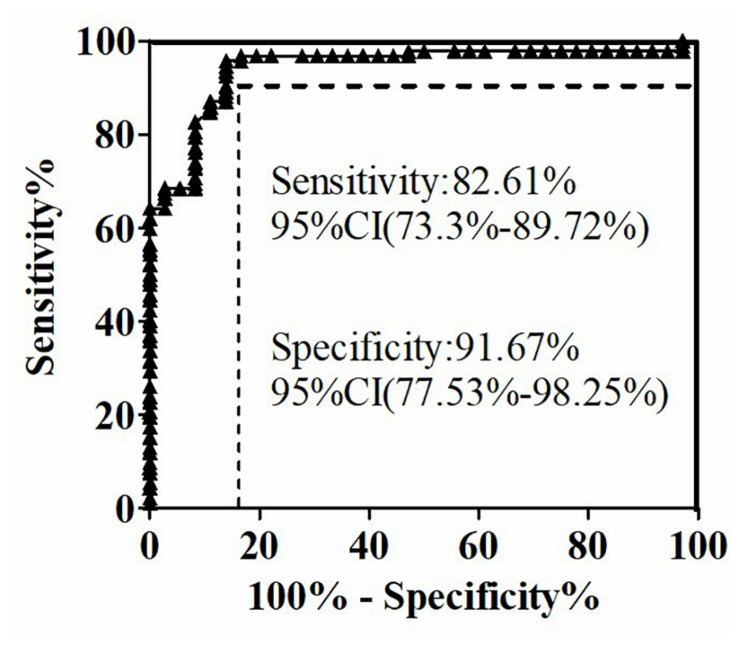
Receiver operating characteristic curve of serum (1,3)-β-D-glucan levels in 128 subjects using the GFαSub252-668 double-sandwich method.

**FIGURE 5:**
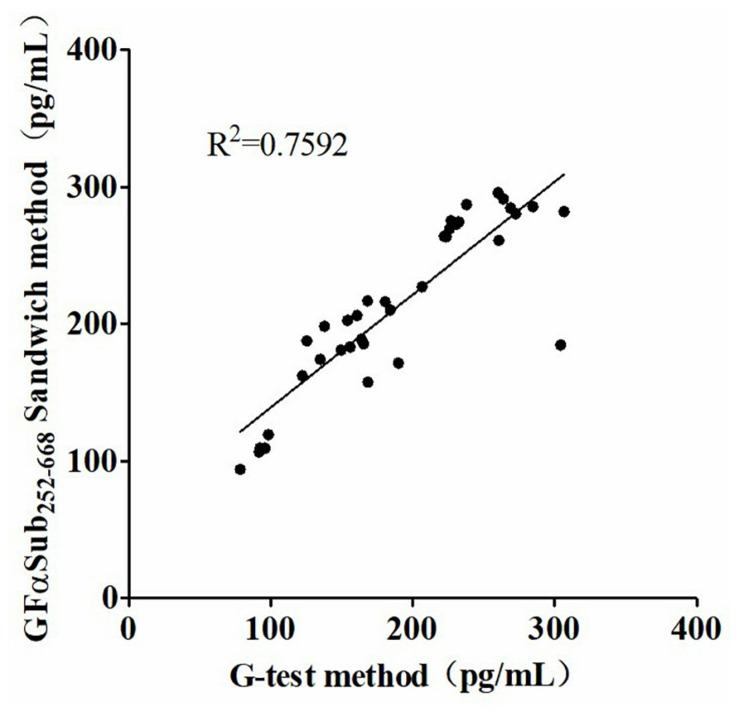
Correlation analysis of serum (1,3)-β-D-glucan levels in 36 patients with invasive fungal disease using the two methods.

The quantitative analysis of (1,3)-β-D-glucan in the samples was performed by establishing a standard fitting curve. The value of R^2^ was 0.9672, and the equation was y = 1/239.8 × x + 0.2586 (Figure 2). Serum samples from 36 patients with pulmonary IFD and 92 patients without IFD were included, and the basic information of the subjects is shown in Tables 1, 2. Of the 128 serum samples tested using the double-sandwich method, the levels of (1,3)-β-D-glucan in patients with IFD were significantly higher than those in patients with bacterial infections and healthy subjects (Figure 3). The measured results were fit using a receiver operating characteristic curve, and the sensitivity and specificity of the GFαSub252-668 double-sandwich method were 91.67% [95% confidence interval (CI): 77.53–98.25%] and 82.61% (95% CI: 73.3–89.72%), respectively. The area under the curve was 0.9423 (95% CI: 0.8999–0.9848) (Figure 4). The levels of serum (1,3)-β-D-glucan were determined in the 36 patients with pulmonary IFD using the two methods, and correlation analysis of the results showed that the value of R^2^ was 0.7592 (*p* < 0.001) (Figure 5).

## Discussion

The clinical course of IFD of the respiratory tract may be severe with poor outcomes. Making a timely diagnosis and initiating appropriate treatment greatly improves survival (Ueda et al., 2009; Shi et al., 2016; Calley and Warris, 2017; Acharige et al., 2018; Ajmal et al., 2018). Clinically, histological analysis is the gold standard, and the G test is more widely used presently (Ueda et al., 2009; Shi et al., 2016). Nevertheless, there are some limitations to these laboratory testing methods, including the low detection rate of fungal culture and smear and the long culture periods. In addition, fungus-specific antibodies in serological examinations cannot fully reflect the infection status. Until recently, the detection of fungus-specific antigens was based on the G test (Ueda et al., 2009; Calley and Warris, 2017; Katragkou et al., 2017; Adachi et al., 2019). In this process, factor G from the serum of *L. polyphemus* is activated by (1,3)-β-D-glucan, which in turn catalyzes the conversion of fibrinogen to fibrin. The assay is highly sensitive for quantifying glucan using dynamic turbidimetric. Nevertheless, the assay principle suggests that it cannot avoid the interference of testing environment, fungal food, drugs, and even some medical equipment. The serum physical properties of patients with abnormalities, such as severe lipidemia, hemolysis, or jaundice, have a substantial impact on the results (Lo Cascio et al., 2015), The present study was designed to immunologically determine the abundance of (1,3)-β-D-glucan in fungal cell walls and to evaluate the status of IFD. Immunoassay can complement the methods described above with its relatively high sensitivity and specificity and can eliminate some interference from other substances in the testing process (Mattos-Graner et al., 2001; Lo Cascio et al., 2015; Anjugam et al., 2018).

Studies have shown that GFαSub derived from marine organisms is efficiently recognizable and binds to glucan with various structures (Ueda et al., 2009; Adachi et al., 2019). In our previous studies, GFαSub from *L. polyphemus*, nematodes, plants, and other species was cloned and expressed, and the results showed that these GFαSub proteins could recognize and bind to (1,3)-β-D-glucan. However, their recognition and binding abilities differed. Subsequently, GFαSub from *L. polyphemus* was selected as the ligand of (1,3)-β-D-glucan based on a comprehensive evaluation of the specificity of recognition and binding capacity. Thus, GFαSub from *L. polyphemus* with these characteristics could be industrialized *via* genetic engineering.

The ID number of the GFαSub provided by the National Center for Biotechnology Information is NM_001314167.1. The full length of the mRNA sequence is 2,007 bp, and it encodes a peptide chain of 668 amino acids. Amino acids 1–20 are signal peptides, 27–253 have glycohydrolase activity, and the rest comprise three glucan-binding domains. Based on this, amino acids 252–668 of the active binding domain of GFαSub252-668 were synthesized and cloned into a prokaryotic expression plasmid and their expression was then induced. After purifying the product, GFαSub252-668 protein was obtained. By constructing a GFαSub double-sandwich system, standard samples and clinical serum samples were tested, and the results showed both high sensitivity and a wide linear range. In the present study, 128 clinical serum samples were tested using this method, and its sensitivity and specificity were 91.67 and 82.61%, respectively, both of which were higher than those of the G test reported in previous studies (Martín-Mazuelos et al., 2015; Lahmer et al., 2016; Shabaan et al., 2018; Ge et al., 2019). The possible reasons for the discrepancy are as follows: (1) the principle of the method: a specific spatial conformation between a ligand and a target is recognized in the GFαSub double-sandwich method, whereas in the G test, the cascade catalytic reaction is activated and initiated by factor G; (2) the quality of the samples: some unknown factors may influence the process of clinical sample collection and delivery that may lead to changes in its characteristics, including severe hemolysis, jaundice, and severe lipidemia; (3) the source of target materials: GFαSub can only bind to glucan with a specific structure; however, the activation of factor G in the G test has no such characteristics, and glucan contained in diets, the environment, drugs, and others may thus interfere with the detection; (4) the protocol of the double-sandwich method in this study is simple, and the entire process is facilitated using an automated enzyme-linked immunoassay instrument that greatly reduces the error. By contrast, all steps of the G test are performed manually, except for the testing step, and the time difference to process samples is likely to cause errors in the final result.

The two methods were subsequently used to determine the amount of (1,3)-β-D-glucan from the serum of 36 patients with IFD, and the correlation analysis of their values showed a good correlation (*R*^2^ = 0.7592). During the analysis, an interesting phenomenon was found in that the levels of serum (1,3)-β-D-glucan in patients with pulmonary Mucor infections (*n* = 6) were slightly lower than in those with yeast-type fungal infections (*n* = 30), which may be because of the difference of (1,3)-β-D-glucan abundance in the cell walls of different species of fungi (Free, 2013). Nevertheless, the method could still be used as an effective means to distinguish IFD.

The findings of the present study showed that our method has a good correlation with the G test. However, because of funding limitations, the G test was performed only in the 36 samples of patients with IFD but not in all the 92 control samples. We will measure the performance of our method in the next stage. Furthermore, HRP was used for signal detection, which limits the sensitivity of our method. In the future, we plan to use luminescent groups for labeling to improve the sensitivity of the detection system.

In summary, we preliminarily tested the application of a specific target *in vivo* to recognize a ligand in clinical testing. This concept provides a solution to the problem of target antibody preparation for small molecular polysaccharides or oligosaccharides, such as (1,3)-β-D-glucan. We hope to develop a new kit for IFD detection that may provide data support for the differential diagnosis of clinical IFD.

## Data Availability Statement

The original contributions presented in the study are included in the article/Supplementary Material, further inquiries can be directed to the corresponding author/s.

## Ethics Statement

The studies involving human participants were reviewed and approved by The First Affiliated Hospital of Chongqing Medical University. The patients/participants provided their written informed consent to participate in this study.

## Author Contributions

JM designed the experiments. FC, PL, and YB performed the experiments and the statistical analysis. FC wrote and edited the manuscript. JM supervised the study. All authors have read and approved the final version of the manuscript.

## Conflict of Interest

The authors declare that the research was conducted in the absence of any commercial or financial relationships that could be construed as a potential conflict of interest.
