# Supplementary material for: A Novel Diagnostic Method for Invasive Fungal Disease Using the Factor G Alpha Subunit From Limulus polyphemus
Source: Front Microbiol. 2021 Jun 28;12:658144. doi: 10.3389/fmicb.2021.658144 (PMC8275026; doi:10.3389/fmicb.2021.658144)
Supplement: Supplementary file 1 [file Data_Sheet_1.DOCX]

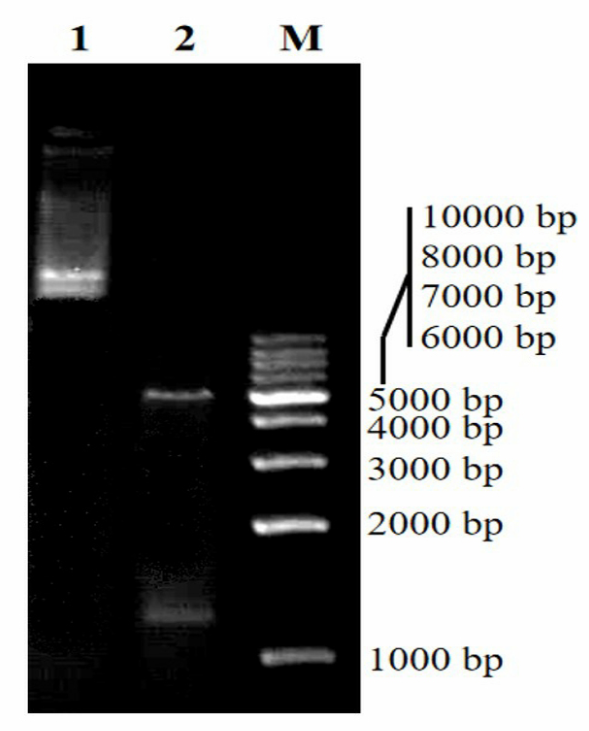


Fig. S1 Identification of recombinant GFαSub expression plasmid by double digestion of restriction enzyme. Lane 1: pET30a-GFαSub_252-668_; Lane 2: pET30a-GFαSub_252-668_ was digested by digestion of Ned I and Hind III; M means DNA marker


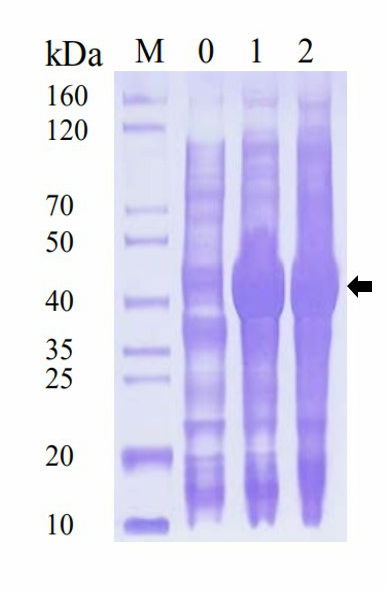


Fig. S2 The expression of recombinant GFαSub_252-668_ was analyzed using SDS-PAGE._._ M = protein marker; Lane 0: the control group with no IPTG induction; Lane 1: induction of IPTG at 25 ℃ for 16 hours; Lane 2: induction of IPTG at 37 ℃ for 16 hours; the arrow shows the position of the recombinant protein of interest.


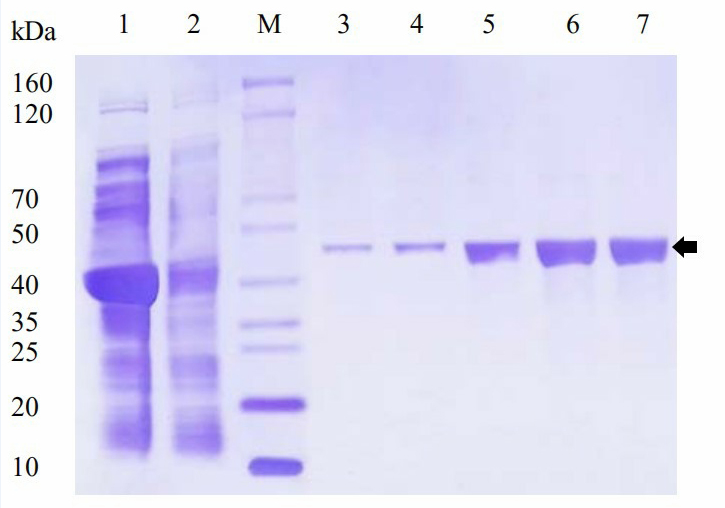


Fig. S3 The purification of recombinant GFαSub_252-668_ was analyzed by SDS-PAGE. M = protein marker; Lane 1: the supernatant of bacterial lysis after induction of IPTG for 16 hours at 25 ℃; Lane 2: the remaining of the bacterial lysis supernatant after IPTG induction and affinity with Ni-NAT; Lane 3: The supernatant collected after washing Ni-NAT with 20 mM imidazole buffer; Lane 4: The supernatant collected after washing Ni-NAT with 30 mM imidazole buffer; Lane 5: The supernatant collected after washing Ni-NAT with 50 mM imidazole buffer; Lane 6: The supernatant collected after washing Ni-NAT with 80 mM imidazole buffer; Lane 7: The supernatant collected after washing Ni-NAT with 200 mM imidazole buffer; the arrow shows the position of recombinant protein of interest.


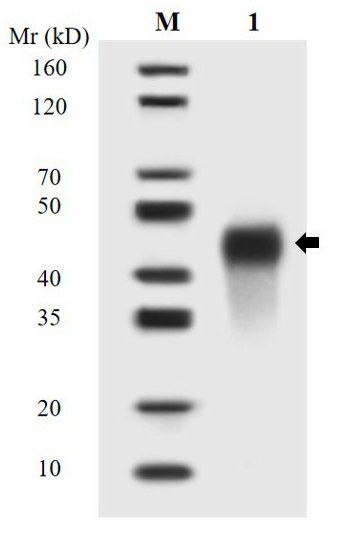


Fig. S4 Purification analysis of the purified recombinant GFαSub_252-668_ by western blot (mouse anti-His tag monoclonal antibody)
